# Extending mechanical thrombectomy service provision to 24/7: a break-even analysis

**DOI:** 10.1186/s12913-024-11290-8

**Published:** 2024-08-07

**Authors:** Joyce S. Balami, Gary A. Ford, Alastair M. Buchan, Alastair Gray, Andrea Francesconi, Paolo Collini, Paolo Candio

**Affiliations:** 1 Acute Stroke Programme, Radcliffe Department of Medicine, University of Oxford, Oxford, UK; 2 Neuroscience Department, Sheffield University Teaching Hospital, Sheffield, UK; 3 Oxford University Hospitals NHS Trust, John Radcliffe Oxford, Oxford, UK; 4 Clinical Pharmacology, University of Oxford, Oxford, UK; 5 Acute Vascular Imaging Centre, University of Oxford, John Radcliffe Hospital, Oxford, UK; 6 Health Economics Research Centre, Nuffield Department of Population Health, University of Oxford, Oxford, UK; 7 Department of Economics and Management, University of Trento, Via Vigilio Inama n. 5, Trento, 38122 Italy

**Keywords:** Break-even analysis, Financial accounting, Health economic perspective, Stroke, Mechanical thrombectomy

## Abstract

**Background:**

Comprehensive stroke centres across England have developed investment proposals, showing the estimated increases in mechanical thrombectomy (MT) treatment volume that would justify extending the standard hours to a 24/7 service provision. These investment proposals have been developed taking a financial accounting perspective, that is by considering the financial revenues from tariff income. However, given the pressure put on local health authorities to provide value for money services, an affordability question emerges. That is, at what additional MT treatment volume the additional treatment costs are offset by the additional health economic benefits, that is quality-adjusted life years (QALYs) and societal cost savings, generated by administering MT compared to standard care.

**Methods:**

A break-even analysis was conducted to identify the additional MT treatment volume required. The incremental hospital-related costs associated with the 24/7 MT extension were estimated using information and parameters from four relevant business cases. The additional societal cost savings and health benefits were estimated by adapting a previously developed Markov chain-based model.

**Results:**

The additional hospital-related annual costs for extending MT to a 24/7 service were estimated at a mean of £3,756,818 (range £1,847,387 to £5,092,788). On average, 750 (range 246 to 1,571) additional eligible stroke patients are required to be treated with MT yearly for the proposed 24/7 service extension to be affordable from a health economic perspective. Overall, the additional facility and equipment costs associated with the 24/7 extension would affect this estimate by 20%.

**Conclusions:**

These findings support the ongoing debate regarding the optimal levels of MT treatment required for a 24/7 extension and respective changes in hospital organisational activities. They also highlight a need for a regional-level coordination between local authorities and hospital administrations to ensure equity provision in that stroke patients can benefit from MT and that the optimal MT treatment volume is reached. Future studies should contemplate reproducing the presented analysis for different health service provision settings and decision making contexts.

## Introduction

Stroke remains the single largest cause of adult disability-adjusted life years lost and the third most common cause of mortality worldwide [1]. Stroke also places a substantial burden on society [2]. A sizeable proportion of stroke survivors will have to give up a productive life and require long-term informal care, affecting families and healthcare systems at large as a result [3]. Most strokes are ischaemic, caused by a blood clot blocking an artery in the brain, depriving it of its blood supply and nutrients, resulting in brain damage. Around 30% of all ischaemic strokes are due to large artery occlusion, although considerable uncertainty surrounds this estimate with studies reporting from 18.6% [4] to 46% [5].

Recent advances in ischaemic stroke treatment have been made, notably, mechanical thrombectomy (MT). This is a minimally invasive surgical procedure which involves the removal of a blood clot in the cerebral arteries to restore the blood flow to the affected brain tissue. MT has been shown to be beneficial for large artery occlusion [6] in improving functional outcomes and reducing disability, in particular if administered within six hours from symptom onset. However, MT has been shown also to provide health benefits within 6–24 h if supported by advanced imaging [7, 8]. Several economic evaluations have been conducted in many countries around the world such as Italy [9], France [10], the United Kingdom [11], China [12], USA [13] and across Europe [14], all broadly reporting favourable cost-effectiveness results compared with standard care.

In the UK, only 3.3% [15] of the conservatively estimated 10% [16] of eligible patients with acute ischaemic stroke are currently treated with MT compared with 5–10% of ischaemic stroke patients receiving MT in Northern and Central Europe [17]. Based on a updated survey conducted in 2021 of 23 active thrombectomy centres in the UK, only four provided MT on a 24/7 basis, with some extending the service window beyond the standard 9.00 to 17.00 routine by a few hours a day [18]. Notably, since that survey more MT centres provide a 24/7 service. Recognizing the time-critical nature of acute ischaemic stroke and its potential health and economic benefits, healthcare professionals and policymakers have therefore endorsed extending this service from standard hours to a fully established 24/7 [19, 20].

To this end, several investment proposals have been put forward by comprehensive stroke centres (CSCs) across England [21]. Putting feasibility issues aside, a 24/7 MT extension will undoubtedly carry a significant opportunity cost in terms of increased need for hospital resources, while at the same time affecting other departments and domains beyond those directly involved with operationalising MT (e.g., emergency department, paramedic team).

Taking a financial accounting approach, hospital managers have developed business cases to assess the affordability of a 24/7 MT extension by considering the expected increase in hospital revenues derived from tariff income [22]. However, local health authorities are increasingly put under pressure to provide value for money services [23]. Hence, a question emerges on whether and under what circumstances such a service provision extension is affordable from a health economic perspective, that is by considering the additional health benefits and societal cost savings generated by treating more patients with MT. This study aims to estimate the required increase in stroke patient volume to treat to offset the additional costs associated with a 24/7 MT extension from such perspective.

## Methods

A break-even analysis is commonly applied to identify the level of revenues at which production costs are offset and therefore the profit is equal to zero, or in other words the benefit-cost ratio is equal to one [24].

### Revenues

To determine the break-even point in this study, this approach was adapted by first defining revenues as the additional patient health-related benefits measured in terms of quality-adjusted life years (QALYs), with one QALY being valued in monetary terms at £20,000 (in the base case scenario), according to the currently accepted willingness to pay threshold for England [25]. We subsequently estimated the per-patient additional number of QALYs generated by treating with MT instead of standard care (based on a previously published cost-effectiveness analysis, details below). By computing the additional costs required for the 24/7 MT extension (see section below) and dividing this value by the per-patient additional number of QALYs generated, we obtained the additional number of stroke patients required to be treated, that is, the break-even point.

The additional QALYs generated by treating eligible stroke patients with MT instead of standard care (i.e., intravenous thrombolysis) were calculated employing a previously published health economic model. More specifically, we adapted a model used in a published cost-effectiveness analysis for the UK, comparing MT with standard care, and using UK-specific, age- and sex-stratified annual eligible stroke cases being obtained from the Global Burden of Disease study [26]. In line with previous studies, a computational approach was applied [27]: starting from an MT volume change value (‘seed value’) corresponding to the median additional number of patients to treat estimated within the analysed business cases (*n* = 350), a break-even point was obtained by dividing the monetary value of the additional QALYs generated by the additional hospital costs.

To align with the business cases and findings from published economic analyses of MT, the time horizon considered was five years, with future costs and benefits being discounted at an annual rate of 3.5% [28]. Costs were expressed at 2021 prices and reported in GBP (£). The health service cost index was applied to account for projected effects of inflation on unit [29].

#### Costs

To reflect the baseline heterogeneity in hospital and service provision settings across England, and consequent heterogeneity in additional resource requirement to transition to a full 24/7 MT service, we analysed four relevant business cases from CSCs which were made available confidentially. These are centres that provide MT to their local population and those from local regional non-CSC areas. The centres vary both in terms of facility and equipment and staff requirements, as well as baseline service provision.

Given the purpose of our study, we simulated a steady-state scenario, where no phasing of the 24/7 implementation was conducted. We estimated the average additional resource requirements, and therefore hospital costs, across the four hospital settings considered based on the respective business cases. Following the classification adopted in published costing guidance for the health sector (NHS England and NHS Improvement, 2023) and previous research studies [9–14], we used a full absorption costing method - that is we considered both fixed and variable costs (Pong, 2006) and grouped hospital-level resource use items in the following three macro categories: staffing, facilities and equipment, and MT operation.

#### Staffing

The 24/7 extension is likely to require an increase in the number of whole time equivalent (WTE) paid staff involved in the MT treatment (fixed or semi-variable costs), in terms of: interventional staff (neuroradiologists and/or neurosurgeons), imaging (radiographers), anaesthesia (consultant and anaesthetic assistant), critical care and stroke/neurologist specialists (consultant) and junior doctors (registrars and/or senior clinical fellows).

#### Facilities and equipment

Depending on the baseline local setting and expected increase in volume of patients to treat with MT, capital investments might be required in the form of additional space and equipment (e.g., biplane angiographic machine). As such, a financial cost of capital based on the interests paid and depreciation and maintenance expenses will need to be accounted for (fixed or semi-variable costs).

#### MT operation

Based on the estimates and classification used in a previous publish study [14], a greater number of operations are going to necessarily absorb a proportional increase (variable costs) in resource use in terms of: devices (stent or catheter), support kits, consumables, anaesthesia drugs and interventional suite time.

#### Non-MT treatment activities

Two hospital functions are likely impacted by a 24/7 MT extension: average length of stay and stroke rehabilitation. As for the former, at least in the short term, a higher number of patients will be treated and consequently be admitted. However, empirical studies have shown that, by reducing mRS score severity, MT reduces the length of hospitalisation and associated need for rehabilitation [30].

### Economic modelling

The baseline CSC scenario was represented by the median hospital and clinical setting across the business cases examined, where MT was assumed to be currently provided, with operational equipment and qualified staff during standard hours (9.00am to 5.00pm) being in place. A previously developed hybrid (decision tree and Markov-based structure) decision-analytic model [14] was used to extrapolate MT effects over the defined time horizon. Briefly, patients who were considered eligible for treatment after experiencing an ischaemic stroke were assumed either to undergo MT or standard care. Patients were categorized into one of seven modified Rankin Scale (mRS) scores at the 3-month mark [31]. These scores range from dead (score of 6) to having no symptoms at all (mRS score of 0). For those who survived the initial three months, the model then projected the risk of death over a span of five years based on their 3-month mRS score, age, and sex. The patient’s QALYs were therefore computed as the product of survival probability and quality of life experienced after the event.

#### Sensitivity analysis

To characterise decision uncertainty [32], we calculated the additional resource requirements under a best- and worst-case scenario, corresponding to the minimum and maximum values across the business case proposals and considered three values for one QALY: £12,936 as suggested by an econometric analysis [33]; £20,000 (lower limit considered by NICE); £30,000 (upper limit considered by NICE) [25]. This produced a three-by-three matrix of values which provided a relevant range of break-even point estimates.

In the UK, a 24/7 MT extension has been commissioned to extend the provision of MT from a local to a regional-level service, where four or more local hospitals are covered. This creates an issue of attribution of the additional capital costs required only for the 24/7 extension. To address this issue, we estimated the total additional costs and respective break-event points under two alternative decision-making scenarios, where additional costs for facilities and equipment were either required or not.

## Results

### Additional hospital costs

On average, the additional annual cost for extending MT to a 24/7 service was estimated at £3,756,818, ranging from a minimum of £2,211,887 to a maximum of £5,092,788. Under a baseline assumption of 350 additional patients required for the investment to be offset by hospital revenues (i.e., financial accounting perspective), the average cost *per additional patient* was calculated at £10,734, ranging from £6,320 to £14,551 per year. Under a scenario of no additional facility and equipment being required for the 24/7 extension, the average cost per additional patient was calculated at £8,617, ranging from £5,278 to £11,359 per year (see Table 1).

**Table 1:** 24/7 extension estimated additional costs (per year)

		annual WTE				attributable cost		
		min	max	mean	unit price	min	max	mean
**Staffing**	Interventional staff	1.20	3.40	2.14	£ 120,000	£ 144,000	£ 408,000	£ 256,400
	Nursing	4.64	14.40	11.00	£ 43,000	£ 199,520	£ 619,200	£ 473,000
	Imaging	2.42	4.80	3.63	£ 46,000	£ 111,320	£ 220,800	£ 166,827
	Anaestetist consultant	0.00	3.37	1.48	£ 120,000	£ 0	£ 404,400	£ 177,600
	Critical care	0.00	1.20	0.40	£ 39,000	£ 0	£ 46,800	£ 15,600
	Stroke consultant	0.00	4.92	3.16	£ 120,000	£ 0	£ 590,400	£ 379,200
	Junior staff	0.50	1.45	1.05	£ 73,000	£ 36,500	£ 105,850	£ 76,650
						**£ 491**,**340**	**£ 2**,**395**,**450**	**£ 1**,**545**,**277**
		annual depreciation / allocation expenses				attributable cost		
		min	max	mean	unit price	min	max	mean
***Facility and equipment***	Angio suite or equivalent (£5.89 m)	0.05	0.10	0.075	£ 5,890,000	£ 294,500	£ 589,000	£ 441,750
	Bi-plane or equivalent (£1.40 m)	0.05	0.10	0.075	£ 1,400,000	£ 70,000	£ 140,000	£ 105,000
	Monitors (operating lease)	0.00	1.00	0.500	£ 23,668	£0	£ 23,668	£ 11,834
	Financial cost of capital investments	0	0.05	0.025	£ 7,290,000	£0	£ 364,500	£ 182,250
						**£ 364,500**	**£ 1,117,168**	**£ 740,834**
		N. units				attributable cost		
		min	max	mean	unit price	min	max	mean
**MT operation**	Interventional suite	1	1	1	£ 810	£810	£810	£810
	Catheter	0.62	0.77	0.72	£ 921	£571	£709	£663
	Stent retriever	0.52	0.63	0.57	£ 3,120	£1,622	£1,966	£1,778
	Support kits	1	1	1	£ 680	£680	£680	£680
	Consumables	1	1	1	£ 191	£191	£191	£191
	Anaesthesia	0	1	0.50	£ 159	£ 0	£159	£80
						**£ 1,356,047**	**£ 1,580,170**	**£ 1,470,707**
						Total annual additional cost		
						min	max	mean
						**£2,211,887**	**£5,092,788**	**£3,756,818**
						Total annual per-patient additional cost		
						min	max	mean
						**£6,320**	**£14,551**	**£10,734**
					**no facility and equipment**	Total annual additional cost		
						min	max	**mean**
						**£1,847,387**	**£3,975,620**	**£3,015,984**
						Total annual per-patient additional cost		
						min	max	**mean**
						**£5,278**	**£11,359**	**£8,617**

### Break-even analysis

On average (that is assuming that a QALY is valued at £20,000 and considering the mean additional costs estimated above), 750 additional eligible patients were found to be required, per year, for the additional cost associated with extending MT to a full 24/7 service to be offset by additional patient’s health and productivity benefits gained (Table 2).

Considering the revenues from tariff income (*n* = 350), the estimated number of additional patients under a health economic perspective is therefore 2.14 times greater than that under a financial accouting standpoint. Under a best-case scenario (a QALY valued at £30,000 and most favourable additional cost estimate), this number drops to 294 and rises to 1,571 under a worst-case hospital costs scenario (a QALY valued at £12,936 and least favourable additional cost estimate). To reach a value of 350 under a best-case additional intervention cost scenario, a QALY would need to be valued at £33,000, that is slightly above the currently accepted threshold upper limit.

Table 2 also shows that the majority of the additional value generated by MT, measured in monetary terms, stems from societal cost savings (on average 59,6%), in particular those relating to the health and social care system. Under a scenario of no additional facility and equipment being required for the 24/7 extension, 602 additional eligible patients were found to be required, on average, per year. This is around 20% of the number required in the base case scenario and drops to 246 and rises to 1,227 under a best-case and a worst-case hospital costs scenario, respectively.

**Table 2:** Reports the results from the break-even and sensitivity analysis conducted

	Per-patient additional benefits	monetary value	Baseline number of patient (*n* = 350) additional benefits	monetary value
Costs	Inpatient	-£ 4,209.25	Inpatient	-£ 1,473,236
	Outpatient	-£ 71.96	Outpatient	-£ 25,185
	Accident & Emergency	£ 1.46	Accident & Emergency	£ 510
	Nursing home	-£ 3,958.25	Nursing home	-£ 1,385,389
	Informal care	-£ 1,558.80	Informal care	-£ 545,580
	Productivity loss	-£ 1,370.60	Productivity loss	-£ 479,710
	*Total cost savings*	£ 11,167.40	*Total cost savings*	£ 3,908,590
QALYs	0.378	£ 7,559.84	0.378	£ 2,645,943
	Total per-patient benefits	£ 18,727.24	Total per-patient benefits	£ 6,554,532
		**Total additional costs**		
		*min*	*max*	**mean**
		£ 8,266,614	£ 19,033,571	£ 14,040,573
	**Additional n. of patients required to break even**			
		*min*	*max*	**mean**
	£ 12,936	682	1,571	1159
	£ 20,000	441	1,016	750
	£ 30,000	294	678	500
			**Total additional costs –** **no facility and equipment**	
		*min*	*max*	**mean**
		£ 6,904,347	£ 14,858,314	£ 11,271,811
	**Additional n. of patients required to break even - no facility and equipment**			
		*min*	*max*	**mean**
	£ 12,936	570	1,227	931
	£ 20,000	369	793	602
	£ 30,000	246	529	401

## Discussion

This paper is concerned with estimating the additional treatment volume required for a 24/7 MT extension to be affordable from a health economic perspective: that is by considering the QALYs gained and societal costs saved by treating more patients with MT instead of standard care. We found that, when a health economic perspective is considered, the required increase in treated patient volume is, on average, around two times that estimated by considering the hospital revenues from tariff income. Under the most favourable hospital setting circumstances this ratio was estimated at 0.84, whereas it reached 4.48 under the worst-case scenario. Overall, the additional facility and equipments cost associated with the 24/7 extension would affect the estimated number of additional patients to be treated by 20%.

To the best of our knowledge, this is the first study directly comparing a financial accounting and a health economic perspective from the benefit side of the argument, to inform an investment decision in health service provision. This study highlights a differential effect in the expected return on investment, favouring a financial accounting perspective, and therefore value recognised by the health care sector as expressed by the reimbursement mechanism of national tariffs, over that estimated from a health-utility and societal cost savings viewpoint. This finding points to a non-negligible misalignment between the two perspectives where, depending on what side of the argument is taken, patient’s health benefits and societal cost savings generated from implementing a clinically superior intervention such as MT are valued up to four and a half times less than that recognised for an MT operation from the Department of Health.

Whereas we adopted a cost-effectiveness principle to conduct this study, in that we compared the costs and health outcomes of two interventions [34], this was not a cost-effectiveness analysis. Though related, affordability and cost-effectiveness are two distinct concepts used to evaluate healthcare interventions: affordability concerns whether the additional costs associated with the intervention are within the financial reach of the payer, whereas cost-effectiveness assesses whether an intervention provides good value for the money spent, considering both its cost and its effectiveness. Several economic evaluations have been conducted clearly establishing that MT is a cost-effective alternative compared to standard care [9–14], and this study does not challenge that body of evidence. The question addressed by this article relates to identifying the conditions (patient volume and resource requirements) under which expanding the health service provision would be affordable from a health economic, instead of from a financial accounting perspective. We employed a break-even analysis approach and comparable examples of such type of economic assessments can be found within the physical activity and obesity literature. Bates et al. (2022) [35] estimated the maximum justifiable cost for a weight loss maintenance intervention for individuals at different BMI and type II diabetes risk, whereas Candio et al. (2023) [36] identified the level of behaviour change required in terms of new regular cyclists for the investment in cycling infrastructure to be sustainable on cost-neutrality grounds.

To estimate the additional patient health benefits generated as a result of a MT treatment, we employed a previously developed health economic model which was based on data from a large cohort study of stroke patients [37] and published regression analyses evaluating the impact of MT on 3-month mRS score and subsequent health care costs, mortality, and quality of life. In addition, we had the opportunity to source hospital resource use information and parameters from four business cases which were made available confidentially. This enabled us to reflect real-world heterogeneity in hospital circumstances and practices and consequently enabled us to adopt a realist approach to evaluation [38], instead of relying on hypothetical scenarios as researchers are often constrained to.

However, our results need to be interpreted in light of some limitations. Whereas we accounted for heterogeneity in hospital baseline settings and therefore resource requirements, we could not formally test for the level of representativeness of our sample for England or other countries. The generalisability of the presented results to different decision-making contexts will indeed depend upon the several modelling assumptions underpinning the analysis and contextual factors, including fluctuations in differential unit prices due to market dynamics, which will induce a further level of uncertainty. We characterised the decision uncertainty associated with the 24/7 MT extension by simulating best and worst-case scenarios and considering three value estimates for a QALY, therefore providing a range of break-even point estimates all of which should provide a realistic range of the additional MT treatment volume required. Nonetheless, we acknowledge that this study is deterministic and essentially exploratory, rather than confirmatory in nature.

Indeed, from an additional cost calculation viewpoint, resource requirements will vary both depending on baseline clinical settings and financing conditions. Avoiding to rely on hypothetical scenarios, this study took a practice-based, real-world perspective by considering what was reported within the business cases made available and simulating six alternative scenarios. In spite of this, residual unobserved heterogeneity in resource requirement may still be present, which limits the generalisability of our study findings accordingly. Nonetheless, our aim was to provide order-of-magnitude timely estimates to inform the current debate on the conditions of affordability of the studied investment decision. We conducted sensitivity analyses to test the robustness of our estimates to variations in resource requirements, including for facility and equipment, and as a result we believe that our analysis highlights the importance for commissioners and providers to understand the broader economic implications of moving to a 24/7 service.

The scope of our evaluation was restricted to a health economic perspective to align with the decision-making context this study aims to inform. One key aspect to consider in interpreting the results presented is the willingness-to-pay threshold. In the UK, the £20,000-£30,000 range is currently used as the decision-making criterion. Hovewer, empirical evidence has indicated that it may be an overestimation of the actual opportunity cost of allocating resources to healthcare interventions instead of other potential uses [33]. Furthermore, other rules apply in other countries and settings. For instance, the World Health Organization has provided guidelines for cost-effectiveness thresholds, suggesting that interventions with an incremental cost-effectiveness ratio of less than 1 to 3 times the GDP per capita are considered cost-effective [39].

Other domains could be considered in evaluating the implementation of a 24/7 MT extension. From the demand side, MT treatment can generate additional benefits including reduced, social care costs and informal care burden. This especially considering a conservative 10% of all stroke patients who would be eligible [40] and could benefit from MT, many of whom currently receive suboptimal treatment due to a lack of 24/7 service provision in the UK. Furthermore, from an ethical standpoint, the NHS should ensure access to MT for patients with acute stroke due to large artery occlusion, comparable to the response of the NHS to major trauma from road accidents and myocardial infarction, both of which are currently provided across the UK on a 24/7 basis.

From the supply side, considering the increased focus from policymakers on developing integrated care models in England and beyond [40], successful implementation and scaling up of a 24/7 service provision will require structural changes to hospital organisation settings and more broadly at the local health authority level. The feasibility of such changes and service delivery will crucially hinge upon a challenging recruitment of the required specialised personnel, as well as re-definition of clinical pathways. In addition, inter-hospital, regional-level coordination will be required for the timely transfer, repatriation of patients and service integration across different levels of the health and social care system [41]. In terms of interventionalists, MT is predominantly provided by interventional neuro radiologists in the UK. But because of staffing shortages, the General Medical Council has recently provided training opportunities for non-radiologist [42]. Indeed, the sharing of resources with other teams providing 24/7 services such as anaesthetic support and increasing capacity are feasible and necessary approaches policymakers should consider.

## Conclusions

Policymakers should consider using these order-of-magnitude estimates to set corresponding additional patient volume targets when planning the extension of 24/7 MT service provision. These findings call for regional-level coordination between hospital managers and local authorities to ensure equity provision in that, within capacity constraints, all eligible stroke patients can benefit from a 24/7 MT service and that the investment is affordable from a health economic perspective. Future studies should contemplate adopting the economic modelling methodology illustrated in this article to establish a pertinent body of evidence for decision makers and reproducing this analysis for different health service provision settings.

## Data Availability

The business case data supporting this study are not publicly available due to commercial reasons.
